# Electroencephalographic monitoring of brain activity during cardiac arrest: a narrative review

**DOI:** 10.1186/s40635-022-00489-w

**Published:** 2023-01-20

**Authors:** Elisabetta Roberti, Giovanni Chiarini, Nicola Latronico, Enrica Chiara Adami, Chiara Plotti, Elisa Bonetta, Federica Magri, Frank Anthony Rasulo, the Coma following Cardiac ArreST study group (COAST)

**Affiliations:** 1 Department of Medical and Surgical Specialties, Radiological Sciences and Public Health, University of Brescia, 25123 Brescia, Italy; 2 Department of Anesthesia, Intensive Care and Emergency, ASST Spedali Civili University Hospital, Brescia, Italy; 3 Cardiothoracic Intensive Care Unit, Cardiothoracic Department, ASST Spedali Civili di Brescia, Brescia, Italy; 4 University of Brescia Residency School in Anesthesiology and Intensive Care Medicine, University of Brescia, Brescia, Italy

## Abstract

**Background:**

To date cardiac arrest (CA) remains a frequent cause of morbidity and mortality: despite advances in cardiopulmonary resuscitation (CPR), survival is still burdened by hypoxic–ischemic brain injury (HIBI), and poor neurological outcome, eventually leading to withdrawal of life sustaining treatment (WLST). The aim of CPR is cardiac pump support to preserve organ perfusion, until normal cardiac function is restored. However, clinical parameters of target organ end-perfusion during CPR, particularly brain perfusion, are still to be identified. In this context, electroencephalography (EEG) and its derivatives, such as processed EEG, could be used to assess brain function during CA.

**Objectives:**

We aimed to review literature regarding the feasibility of EEG and processed or raw EEG monitoring during CPR.

**Methods:**

A review of the available literature was performed and consisted of mostly case reports and observational studies in both humans and animals, for a total number of 22 relevant studies.

**Results:**

The research strategy identified 22 unique articles. 4 observational studies were included and 6 animal testing studies in swine models. The remaining studies were case reports. Literature regarding this topic consists of conflicting results, containing studies where the feasibility of EEG during CPR was positive, and others where the authors reached opposite conclusions. Furthermore, the level of evidence, in general, remains low.

**Discussion:**

EEG may represent a useful tool to assess CPR effectiveness. A multimodal approach including other non-invasive tools such as, quantitative infrared pupillometry and transcranial Doppler, could help to optimize the quality of resuscitation maneuvers.

## Take home message

Future randomized trials are required to adopt EEG monitoring during CPR, also considering its possible role in a multiparametric assessment of cerebral function and perfusion.

## Introduction

### Rationale

CA represents nowadays the third leading cause of death in Europe [1]. A distinction is usually made between in-hospital cardiac arrest (IHCA), with a European annual incidence of 1.5/2.8 per 1000 hospital admissions, and out-of-hospital cardiac arrest (OHCA), where the true incidence is not known due to substantial underreporting and excessive variability between countries: it is estimated to be between 67 and 170 per 100,000 inhabitants [1].

According to the EuReCa TWO study, the mean age of patients who undergo resuscitation is 67.6 years, 65% are male, while in those for whom CPR is not started the mean age is 71.5 years [2]. Only approximately 20% of the patients have an initial shockable rhythm [2].

Early initiation of CPR, and increased use of Automated External Defibrillators (AEDs) have improved the chance of CA survival. The EuReCa TWO study reported an overall survival rate of 8% after OHCA in Europe [2], while for IHCA the chance of survival to discharge/30 days varies from 15 to 34% [1].

The main cause of death is active WLST after return of spontaneous circulation (ROSC) whenever the severity of HIBI indicates a poor neurological outcome [3]. HIBI is frequent in CA patients and its pathophysiology follows a two-hit model [4]: first, the reduction of cerebral blood flow, followed after ROSC by ischemia–reperfusion injury [4].

Accurate prognostication is essential to prevent inappropriate WLST, and to avoid futile treatment in patients with irreversible neurological injury [5]: besides overall survival, post-CA neurological status and subsequent patient’s quality of life should be considered as measures of CPR outcome. In order to prevent HIBI, CPR should maintain adequate cerebral function until cardiac pump recovers.

In this context, early neurological monitoring during and after CPR can provide prognostic clinical features of pivotal significance in post-resuscitation care.

The 2021 European Resuscitation Council (ERC) and European Society of Intensive Care Medicine (ESICM) guidelines suggest that the prognostication strategy algorithm should be applied to all patients with an absent, extensor or abnormal flexion motor response to pain after ≥ 72 h [3]. In such cases, a poor outcome is likely if two or more of the following conditions occur: *(a)* no pupillary and corneal reflexes at ≥ 72 h; *(b)* bilaterally absent N20 somatosensory evoked potential (SSEP) wave at ≥ 24 h; *(c)* highly malignant EEG at > 24 h; *(d)* neuron specific enolase (NSE) > 60 µg/L at 48 h and/or 72 h; *(e)* status myoclonus 72 h; *(f)* or a diffuse and extensive anoxic injury on brain CT/MRI [3]. However, these different conditions only account for prognosis following CPR.

Factors that could affect neurological outcome during CPR are yet to be clearly identified. To date, the best predictor of outcome is end-tidal carbon dioxide (EtCO_2_) level after 20 min of resuscitation [6]. However, using EtCO_2_ thresholds alone for decision-making to stop resuscitation is not recommended, due to the absence of blind studies and the subsequent risk of self-fulfilling prophecy [6]. Furthermore, although EtCO_2_ might be a surrogate marker for systemic circulation status, it does not account for cerebral perfusion pressure (CPP) [7].

CPP is derived from mean arterial pressure (MAP) and intracranial pressure (ICP), which is invasively measured using a catheter inserted through the skull and the dura mater. This hinders the possibility of acquiring ICP measurements while performing CPR.

Conversely, EEG offers a continuous real-time non-invasive monitoring of brain function, and is sensitive to cerebral blood flow (CBF) changes [8]. Traditionally, EEG has been used to assess the neurological function after CA since the 1960s, when Hockaday et al. proposed a grading system to categorize EEG patterns and the related prognosis [9]. In most cases suppressed patterns are found immediately after CA occurs, progressively increasing in amplitude and continuity during the recovery phase [10]. Therefore, EEG gains reliability in determining prognosis only after 24 h.

However, EEG sensitivity to CBF variations might be useful to predict the adequacy of cerebral oxygenation during chest compressions: few seconds after cardiac arrest, the EEG becomes isoelectric [9]; while after CPR initiation, a return of signal activity might represent a sign of cerebral perfusion [11].

Furthermore, it might be possible to use raw EEG, such as Bispectral Index™ (BIS™) or SedLine® PSi, initially developed to monitor the depth of anesthesia. These methods are based on the signal registered by frontal electrodes, in a more rapid and easier way than positioning EEG electrodes [12]. This characteristic may be convenient, considering that the rare adoption of neurophysiologic monitoring during resuscitation is partly related to the fear of delays in starting CPR or increasing interruptions in chest compressions.

Other possibilities to assess cerebral perfusion and function are transcranial Doppler, optic nerve sheath diameter evaluation, and quantitative infrared pupillometry [13]. Among these non-invasive tools, quantitative infrared pupillometry is particularly promising [14], and easy to perform. Neurological Pupil index (NPi) is the one of the most important evaluating parameters since it is independent of pupil size and sedation [15]. Moreover, an NPi ≤ 2 correlates with poor neurologic outcome in OHCA with a predictive value of 100% [13, 17].

### Objectives

The goal of this review is to discuss the feasibility of EEG monitoring during CPR through analysis of the data available in literature.

## Methods

Given the lack of published literature, we did not perform a systematic review.

### Search strategy

A literature search was performed with MEDLINE via PubMed and EMBASE databases. No restrictions were placed on date, language, or country of study publication.

### Study selection

We searched through PubMed using the following medical subjects heading (MeSH) terms: ‘cardiac arrest’ AND ‘cardiopulmonary resuscitation’ AND ‘electroencephalogram’. This yielded 127 publications. Moreover, we used the following Emtree terms for EMBASE: ‘heart arrest’ AND ‘resuscitation’ AND ‘electroencephalogram’. This yielded to 573 results.

After duplicates were removed, we identified 22 eligible studies, including only those considering EEG or raw EEG as neuro-prognostication markers during CPR, among which 6 are conducted in animals, particularly in swine models of cardiac arrest. As a matter of fact, pigs represent a valid model of neuro-prognostication during cardiac arrest, as not only cardiovascular physiology is similar in swine and humans, but also the gyrated pig brain shares affinity with the gyrated primate brain [16].

Table 1 lists human studies and respective prognostication markers, while Table 2 lists the studies conducted in animals.

**Table 1:** EEG, raw EEG, and Bispectral index as CPR effectiveness markers

Study	Study design	Prognostication markers	Conclusions
Young et al. [19]	Case report	EEG^1^	Possible use of EEG during CPR^2^
Losasso et al. [11]	Case report	Raw EEG^1^	EEG might be effective in predicting CPP^3^ and oxygenation during CPR^2^
Chakravarthy et al. [20]	Case report	BIS™^4^	BIS™^4^ monitoring might confirm the adequacy of CPR^2^, targeting a value of about 40
Kluger [21]	Case report	BIS™^4^	- BIS™^4^ might be a surrogate of CBF^5^ - Possible EMG^6^ interference
Szekely et al. [22]	Case report	BIS™^4^	High BIS™^4^ value reflects cerebral activity
Azim et al. [12]	Case report	BIS™^4^	BIS™^4^ might be useful during cardiopulmonary arrest
Fatovich et al. [24]	Observational study (21 patients)	BIS™^4^	BIS™^4^ is not a clinically reliable marker of CPR^2^ efficacy due to mechanical artifacts induced by chest compressions
Sethi et al. [27]	Case report	EEG^1^	CPR^2^ artifact consists of periodic sharply contoured waveforms
Chollet-Xémard et al. [25]	Observational study (92 patients)	BIS™^4^	BIS™^4^ monitoring during resuscitation did not predict ROSC^7^
Nitzschke et al. [26]	Case report	Raw EEG^1^	EEG^1^-monitoring is not a useful tool during CPR^2^ due to artifacts related to chest compressions
Reagan et al. [18]	Observational study (16 patients)	Sedline PSi^8^	Real-time monitoring of cerebral oxygenation and function during CPR^2^ is feasible
Biesbroek et al. [23]	Case report	EEG^1^	EEG^1^ activity returned several seconds after initiation of chest compressions, reflecting an increase in cerebral perfusion
Nguyen et al. [28]	Case report	PSi^8^	EEG^1^-based monitors may be useful during cardiac arrest
Jung et al. [29]	Case report	BIS™^4^	BIS^4^ monitoring might be used as a reference device to determine whether CPR^2^ should be continued
Soto et al. [30]	Observational study (16 patients)	BIS™^4^	BIS values during CPR do not predict survival in cardiac arrest
Dumans-Nizard [31]	Case report	BIS™^4^	BIS™^4^ monitoring may be misleading

^1^EEG = electroencephalography; ^2^CPR = cardiopulmonary resuscitation; ^3^CPP = cerebral perfusion pressure; ^4^BIS™ = Bispectral index; ^5^CBF = cerebral blood flow; ^6^EMG = electromyography; ^7^ROSC = return of spontaneous circulation; ^8^PSi = Patient State index

**Table 2:** EEG as a neuro-prognostic marker in animal models

Study	Prognostication markers	Conclusions
Kim et al. [32]	- EEG^1^ - Carotid blood flow	Time-domain magnitude and entropy measures of EEG^1^ correlated with the change of CBF^2^
Kim et al. [7]	EEG^1^	An EBRI^3^ model showed excellent performance
Kim et al. [33]	EEG^1^	Non-invasive EEG^1^-based automated screening of CBF^2^ recovery has the potential for real-time feedback of cerebral circulation during CPR^4^
Choi et al. [34]	- EEG^1^ - EtCO_2_^5^	EBRI^3^ was successfully obtained during resuscitation and had a statistically moderate correlation with EtCO2^5^
Caballero et al. [35]	BIS™^6^	BIS monitoring values showed correlation with values of mean arterial pressure and EtCO2^5^
Rees et al. [36]	BIS™^6^	The product of EtCO2 and BIS is correlated with cerebral perfusion pressure

^1^EEG = electroencephalography; ^2^CBF = cerebral blood flow; ^3^EBRI = EEG-based brain resuscitation index; ^4^CPR = cardiopulmonary resuscitation; ^5^EtCO2 = end-tidal carbon dioxide; ^6^BIS™ = Bispectral index

**Fig. 1:**
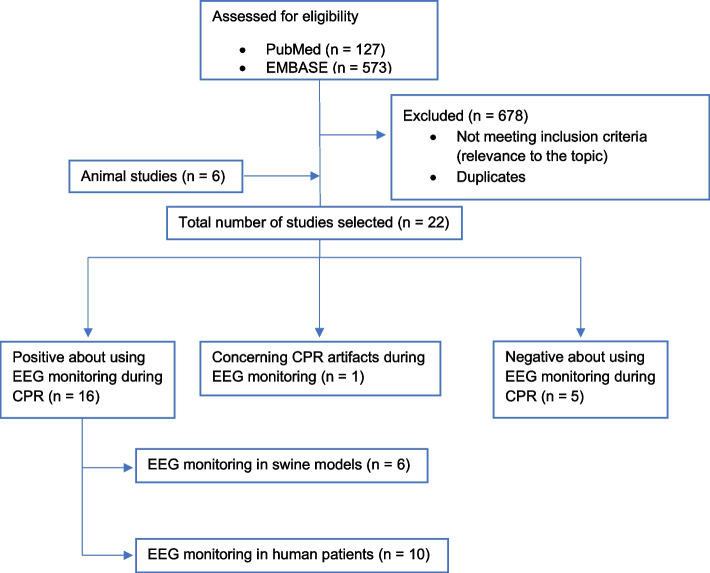
Literature search process

Figure 1 illustrates literature search process.

## Results

The search strategy identified 22 unique articles (Tables 1, 2). Dates of publication ranged from 1985 to 2022. 3 observational cross-sectional studies and 1 observational prospective study were included (145 patients—Table 1), and 6 (27.3%) animal testing studies in swine models. The remaining were case reports.

16 studies among the total number of 22 regarded human patients and mostly raw EEG was used to predict CPR efficacy, while EEG use was essentially limited to cardiac arrest in the perioperative period.

One study associated EEG monitoring using SedLine® technology with real-time cerebral oxygenation monitoring [18], observing that cortical activity was only present at regional cerebral oxygen saturation (rSO_2_) levels above approximately 20%. At rSO_2_ levels equal or inferior to 19% and soon after CA occurs, a voltage suppression pattern was consistently detected. Other observed patterns were theta background activity, delta background activity, periodic discharges, burst suppression, spike and wave, and rhythmic delta activity [18]. Delta background activity was detected only at rSO_2_ levels above 40%, possibly reflecting a better neurological outcome [18].

Ten of the 16 human studies reported that EEG/raw EEG monitoring during CPR, as a marker of cerebral function, is feasible [11, 18–25, 28, 29], while 5 expressed a negative opinion [24–26, 30, 31], highlighting the risk of mechanical artifacts due to external chest compressions. This concern was specifically analyzed in another case report study [27], which showed CPR artifacts as periodic sharply contoured waveforms. Of interest, as compressor fatigue takes over, CPR artifacts may vary [27]. Moreover, in this case, an isoelectric pattern was noted between CPR attempts, possibly reflecting absence of cortical activity [27].

As for animal models [7, 32–36], ventricular fibrillation was medically induced and treated following a standard protocol. In all these cases, EEG showed a good correlation with cerebral blood flow recovery. In 2 (50%) [7, 34], EEG signal was used to derive a parameter called EEG-based brain resuscitation index (EBRI) that could be used as surrogate of cerebral perfusion pressure [7], and also showed moderate correlation with EtCO_2_ [34].

## Discussion

Despite recent advances in performing CPR, CA remains a frequent cause of death. Although CPR might be effective in restoring systemic circulation, single organs could suffer persistent damages secondary to hypoxia and ischemia.

**Fig. 2:**
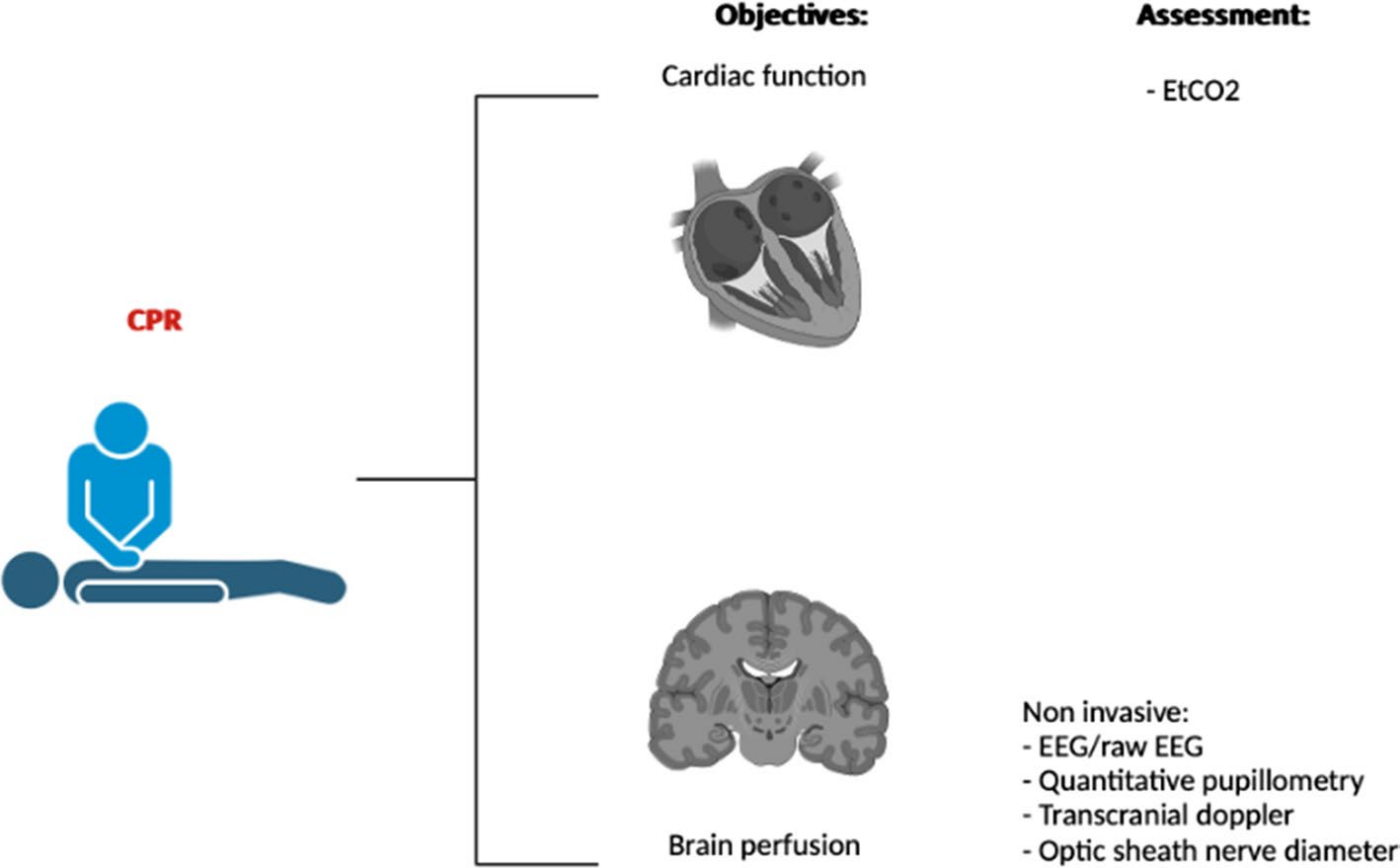
Cardiopulmonary resuscitation (CPR) should aim to make up for the cardiac pump, maintaining blood flow to target organs, such as brain. Thus, CPR performance should be assessed not only through markers of systemic circulation, such as EtCO_2_

Particularly, brain damage has a significant impact in patients. The aim of CPR should be to restore cardiac function, and at the same time preventing brain injuries due to the low/no-flow state (Fig. 2).

Early recognition of a patient who may neurologically recover after CPR still represents a challenge. The actual prognostication algorithm is applicable only at ≥ 72 h from ROSC [3].

At present there is not a single parameter that can estimate the quality of cerebral perfusion and the possible success of resuscitation maneuvers on brain function [6].

Monitoring cerebral function during CPR could provide information on prognosis and guide decision-making algorithm.

In this context EEG may be a non-invasive and economic method to monitor cerebral function. Changes in the EEG pattern in relation to brain hypoxic/ischemic damage have long been recognized: just after CA occurs EEG becomes isoelectric, after CPR beginning EEG pattern might change, due to cerebral reperfusion. Particularly, a delta background activity, being associated with higher rSO_2_ values, might forecast better neurological outcome [18].

However, the complexity of electrode placement and interpretation, limited EEG monitoring during CPR to very few case reports or human studies. In this context, the advent of simplified EEG monitoring systems, such as processed EEG, which are gradually increasing in reliability through the years, may allow to analyze cortical brain activity. This information could be added to the automated pupillometry monitoring results derived from brainstem activity, thus potentially providing a global picture of the brain’s status during CPR.

While results in animal models are promising [7, 32–36], literature data in humans are still controversial. Few studies suggested that BIS™ or PSi values, and/or EEG analysis might reflect cerebral oxygenation [11, 17–23]; nevertheless, others showed that BIS™ values, and EEG monitoring are not reliable indicator of cerebral function due to mechanical artifacts related to external chest compression and involuntary head movements [26]. Thus, after reviewing the literature, we have not found any convincing evidence suggesting standardized use of simplified EEG during CPR. However, use of new and easy-to-use/apply devices, such as Bluetooth headsets and casques, along with the evolution of automated chest compression technique, may help in standardizing the signal.

## Limitations

Our narrative review is limited by the low level of evidence of the available literature. Most of the studies in human patients, in fact, are case reports. As for animal studies, the prediction models should be regarded as preliminary.

## Conclusions

Neurological prognosis after CA is still a challenge. The use of EEG, and raw EEG models has evolved: at the moment, its role is limited to post-resuscitation care. Innovative EEG methodologies, including quantitative analysis, and machine learning, seem promising for assisting clinicians during the CPR decision-making process, despite the conflicting literature.

Although further studies in humans are needed to explore the potential usefulness of different EEG tools, neuro-monitoring could optimize resuscitation identifying individuals in whom further resuscitation attempts might reveal futile. Nevertheless, in the absence of evidence-based studies, EEG patterns during CPR are not yet recommended to support withdrawal decisions.

## Data Availability

The authors confirm that the data supporting the findings of this study are available within the article.
